# Cacao polyphenols regulate the circadian clock gene expression and through glucagon-like peptide-1 secretion

**DOI:** 10.3164/jcbn.20-38

**Published:** 2020-06-05

**Authors:** Ken-yu Hironao, Yuji Mitsuhashi, Shujiao Huang, Hideaki Oike, Hitoshi Ashida, Yoko Yamashita

**Affiliations:** 1 Department of Agrobioscience, Graduate School of Agricultural Science, Kobe University, 1-1 Rokkodai-cho, Nada-ku, Kobe 657-8501, Japan; 2 Food Research Institute, National Agriculture and Food Research Organization (NARO), Tsukuba, Ibaraki 305-8642, Japan

**Keywords:** circadian clock, glucagon like peptide-1, cacao liquor procyanidin, AMPK, liver

## Abstract

Energy metabolism and circadian rhythms are closely related together, i.e., the timing of nutrient intake affects metabolism under the regulation of circadian rhythms. Previously, we have reported that cacao liquor procyanidin (CLPr) promotes energy metabolism, resulting in preventing obesity and hyperglycemia. However, it is not unclear whether CLPr regulates clock gene expression. In this study, we investigated whether the administration timing of CLPr affected clock gene expression and found that CLPr regulated the circadian clock gene expression through the glucagon-like peptide-1 (GLP-1) signaling pathway. CLPr administration at Zeitgeber time 3 increased the expression level of *Per* family and *Dbp* in the liver. At the same administration timing, CLPr increased GLP-1 and insulin concentration in the plasma and phosphorylation of AMPK in the liver. It was noteworthy that an antagonist for GLP-1 receptor Exendin (9-39) canceled CLPr-increased expression of *Per* family and *Dbp* and phosphorylation of AMPK in the liver, in addition to insulin secretion. These results strongly suggest that CLPr-induced GLP-1 regulates the changes in clock gene expression in the liver through increased insulin. Thus, CLPr is a possible functional food material for prevention and/or amelioration of metabolic disorders through preventing circadian disruption through GLP-1 and AMPK pathways.

## Introduction

All animals and plants have 24-h circadian rhythm to synchronize biological functions for adapting to the environmental changes. The molecular oscillator of circadian clock is regulated by the transcription/translation feedback loops consisting of heterodimeric transcription factors, brain and muscle arnt-like 1 (BMAL1), circadian locomotor output cycles kaput (CLOCK) and their repressors, cryptochromes (CRY) and period (PER).^(1)^ In mammalian, central clock is located in the hypothalamic suprachiasmatic nucleus that receives light and regulates peripheral clocks throughout the organs via hormonal and autonomic neural pathways.^(2)^ Peripheral clocks are expressed in all tissues and mainly modulated by breakfast.^(3)^ Keeping the correct rhythm of clock gene expression, amplitude and phase shift, are important for maintaining human health.

Clock genes are known to regulate not only circadian rhythm but also energy metabolism.^(4–6)^ Abnormalities in clock genes relate to disorders of energy metabolism and cause metabolic syndrome. Clock genes also regulate hormone secretion.^(7–10)^ For instance, insulin secretion exhibits a circadian pattern in human pancreatic β-cells.^(11)^ It was reported that *Bmal1*- and *Clock*-gene knockout mice lead to lack rhythmicity of insulin activity patterns.^(12)^ Recently, it has been reported that glucagon-like peptide-1 (GLP-1) secretion from intestinal L-cells after food ingestion also has circadian rhythms.^(13)^ Binding of GLP-1 to its receptor in pancreas, resulting in the induction of insulin secretion to maintain the blood glucose homeostasis. In contrast, insulin and GLP-1 also regulate the clock gene expression. For example, insulin is possible to reset clock phase in cultured hepatocytes.^(14)^ Insulin also promotes Akt-mediated phosphorylation of BMAL1 at Ser42, which leads to the reduction of BMAL1 nuclear accumulation to reset hepatic circadian rhythms.^(15)^ Food intake and insulin injection cause the up-regulation of *Per2* mRNA and down-regulation of *Rev*-*erbα* mRNA within 2 h in normal mice, whereas these changes are not observed in streptozotocin-treated insulin-deficient mice.^(16)^ Therefore, insulin is a key factor for regulating circadian rhythm, but it is still unclear how hyperinsulinemia alters the expression of clock genes in the liver. It is also reported that a GLP-1 receptor agonist Exendin-4 affects the *Per1* clock gene mRNA level in the liver 12 h after the administration,^(17)^ though the underlying molecular mechanism is unclear yet. These hormones and expression of peripheral clock genes affect each other, i.e., circadian clock genes and metabolism have reciprocal relationship. Therefore, clock genes play an important role in the regulation of metabolism related to maintaining homeostasis.

Not only nutrients but also food factors, especially polyphenols, have been noted to affect clock genes. Recent reports have demonstrated that polyphenols can adjust and/or regulate the circadian rhythm. For example, resveratrol affects the circadian rhythm of *Sirt1* expression to restore the clock genes in Rat-1 fibroblast cells. In mice, resveratrol restores the circadian rhythmic disorder of lipid metabolism induced by the intake of high-fat diet.^(18,19)^ Proanthocyanidins have the ability to modulate peripheral molecular clocks in both healthy and obese rats.^(20)^ It is recently reported that (–)-epigallocatechin-3-gallate, a major catechin in green tea,^(21)^ ameliorates diet-induced metabolic syndrome associating with the circadian clock.^(22)^ Palmitate could alter the clock genes through GLP-1 secretion in GLUTag L-cells.^(23)^ These results prove that polyphenols have a potential to affect metabolisms through altering the rhythms of peripheral clocks. It is, however, poorly reported that the effects of ingestion timing of polyphenols on clock gene expression. Ingestion of caffeine at night, but not at morning, delayed the rhythms,^(24)^ though its underlying mechanism is still unclear.

In this study, we focused on cacao polyphenols which are reported to have various health beneficial effects. Cacao liquor procyanidin (CLPr), which is an extract from cacao liquor containing (+)-catechin, (–)-epicatechin and procyanidins abundantly, prevents hyperglycemia by stimulating glucose uptake and glucose transporter type 4 translocation through AMP-activated protein kinase (AMPK) pathway in muscle cells.^(25,26)^ CLPr increases energy metabolism and prevents obesity and hyperglycemia through AMPK pathway.^(26)^ Moreover, CLPr activates enteroendocrine GLP-1/insulin pathway to reduce the postprandial hyperglycemia. Oral intake of cinnamtannin A2, a tetrameric procyanidin contained in CLPr, increases the GLP-1 and insulin secretions in mice.^(27)^ From these results, we hypothesize that CLPr regulates the circadian clock gene through GLP-1/insulin and AMPK pathways. In this study, we investigated the effects of CLPr on the expression of circadian clock genes in mice by focusing on the function of GLP-1.

## Materials and Methods

### Reagents

Plasma glucose, GLP-1, insulin and adiponectin levels were measured using the corresponding commercial assay kit [Mouse Insulin Elisa Kit (RTU) (Akrin-011RU) and GLP-1 (Active) Elisa Kit (Akmgp-011) were purchased from FUJIFILM Wako Shibayagi Co. (Gunma, Japan), while Lab assay Glucose kit was from FUJIFILM Wako Pure Chemical Co. (Osaka, Japan)]. Exendin (9-39) was obtained from R&D Systems, Inc. (Minneapolis, MN). Antibodies against AMPK, p-AMPK liver kinase B1 (LKB1), p-LKB1 and p-Ca^2+^/calmodulin-dependent protein kinase kinase (CaMKK) 2 and β-actin (Cell Signaling Technology Inc., Beverly, MA) were used in this study. Antibody against CaMKK2 was purchased from Abcam (Hercules, CA). All other reagents used were of the highest grade available from commercial sources.

### Polyphenol composition of CLPr

CLPr was prepared from cacao liquor as previously reported and kindly provided from Meiji Holdings Co., Ltd. (Tokyo, Japan).^(28)^ Polyphenols in CLPr were quantified by a high performance liquid chromatography and liquid chromatography-mass spectrometry as previously described.^(28,29)^ Polyphenol composition of CLPr is shown in Table 1 and the amounts of individual polyphenol are represented as epicatechin equivalent. The total amount of polyphenol was separately measured by the Prussian blue method.^(30)^

### Animal treatment

All animal experiments were approved by the Institutional Animal Care and Use Committee (Permission #27-05-08) and carried out according to the guidelines for animal experiments at Kobe University. Male C57BL/6N mice (5 weeks old) were obtained from Japan SLC, Inc. (Shizuoka, Japan), and kept in a temperature-controlled room (22 ± 2°C) with a 12:12-h light/dark cycle [lights on at 8:00 am: equal to Zeitgeber time (ZT) 0]. Mice were acclimatized for 7 days with free access to a D10012M (AIN-93M base) diet (Research Diets, Inc., New Brunswick, NJ) and tap water.

Experiment 1: After acclimatization, C57BL/6N mice were randomly divided into 8 groups of 5 each. The mice were orally administrated CLPr at 150 mg/kg body weight or water (5 ml/kg body weight) as a control at ZT-3, 3, 9 and 15. After 3 h-administration, the mice were sacrificed under anesthesia using sevoflurane as an inhalational anesthetic and sodium pentobarbital as an analgesic. Liver, skeletal muscle and adipose tissue were taken and used to measurement of the expression level of clock genes by RT-PCR.

Experiment 2: Forty C57BL/6N mice were also randomly divided into 8 groups of 5 each. Twenty mice of them were orally administrated CLPr at 150 mg/kg body weight or water (5 ml/kg body weight) at ZT 3. A GLP-1 receptor antagonist Exendin (9-39) was intraperitoneally injected to the mice at 200 nmol/kg body weight 5 min before the CLPr administration. After 1 h-CLPr administration, the mice were sacrificed under the same anesthesia conditions and blood was collected from cardiac puncture in a heparinized microtube. Plasma was obtained by centrifugation at 5,000 × *g* for 10 min at 4°C. Plasma level of GLP-1 and insulin was measured by using corresponding commercial kit according to the manufacturer’s instructions. Liver was collected to use for measurement of phosphorylation of LKB1 and AMPK by western blotting. Another twenty mice were also dived into the same groups and given the combination of CLPr and/or Exendin (9-39). They were sacrificed 3 h after the CLPr administration and liver was collected to measure the expression level of clock genes and energy metabolism-related genes by RT-PCR.

### Analysis of mRNA by RT-PCR

Isolation and purification of mRNA and preparation of cDNA were performed as previously described.^(26)^ cDNA was subjected to RT-PCR amplification using SYBR Green premix Taq (Takara Bio, Shiga, Japan). The forward and reverse primers are listed in Table 2. Reactions were run in a real-time PCR system (TaKaRa PCR Thermal Cycler Dice, Takara Bio). Relative gene expression level was calculated by the comparative CT method, using the expression of the *Gapdh* gene as an internal control.

### Western blot analysis

Liver lysate was prepared according to the previous report.^(26)^ After protein concentration in the lysate was quantified by a Lowry’s method,^(31)^ the lysate was subjected to Western blot analysis following the sodium dodecyl sulfate-polyacrylamide gel electrophoresis using a 10% gel. The proteins were transferred to a polyvinylidene difluoride membrane (Merck Millipore Ltd., Billerica, MA) and the membrane was treated with commercially available blocking solution (Blocking One, Nacalai Tesque, Kyoto, Japan) for 1 h at room temperature. The membrane was incubated with antibodies against, p-AMPK (1:5,000), AMPK (1:5,000), p-LKB1(1:5,000), LKB1, CAMKK and β-actin (1:10,000) overnight at 4°C, followed by the corresponding horseradish peroxidase-conjugated secondary antibody (1:20,000) for 90 min at 4°C. The proteins bands were visualized using Immuno Star LD (FUJIFILM Wako Pure Chemical Co.) and detected with a light-Capture II (ATTO Co., Tokyo, Japan). Density of the specific band was determined using ImageJ software (National Institutes of Health, Bethesda, MD).

### Statistical analysis

**Fig. 1:**
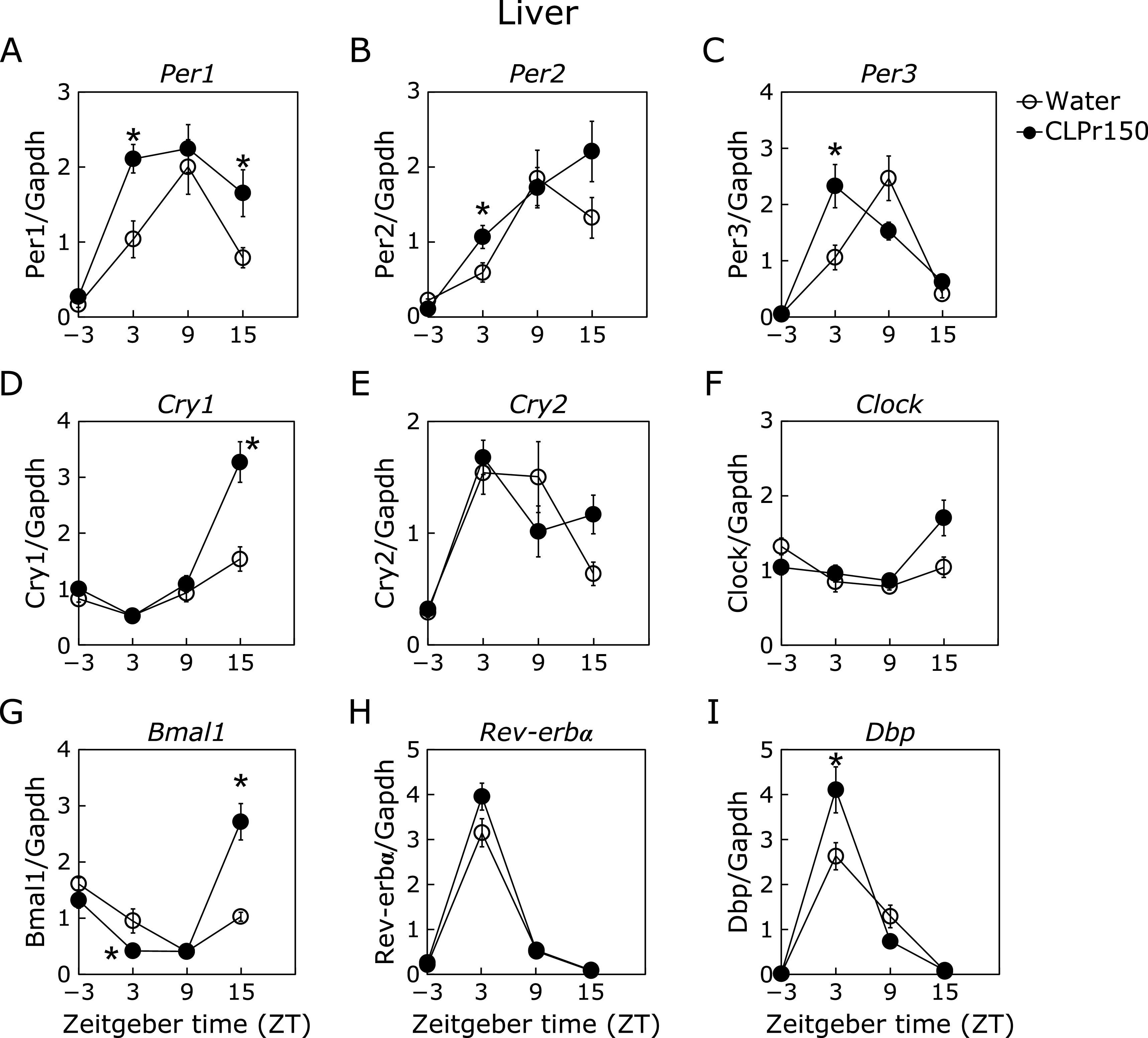
Expression of clock and energy metabolism-related genes in the liver 3 h after the administration of CLPr. Mice were orally administered CLPr at 150 mg/kg body weight or water (5.0 ml/kg body weight) at ZT-3, 3, 9 or 15. The expression level of clock genes was measured in the liver and their mRNA level was normalized by *gapdh*. The polygonal line graphs for *Per1* (A), *Per2* (B), *Per3* (C), *Cry1* (D), *Cry2* (E), *Clock* (F), *Bmal1* (G), *Rev-erbα* (H) and *Dbp* (I) are shown. Data are represented as the means ± SE (*n* = 5). *Significantly different from the control group at each time point (*p*<0.05; Dunnett’s test).

**Fig. 2:**
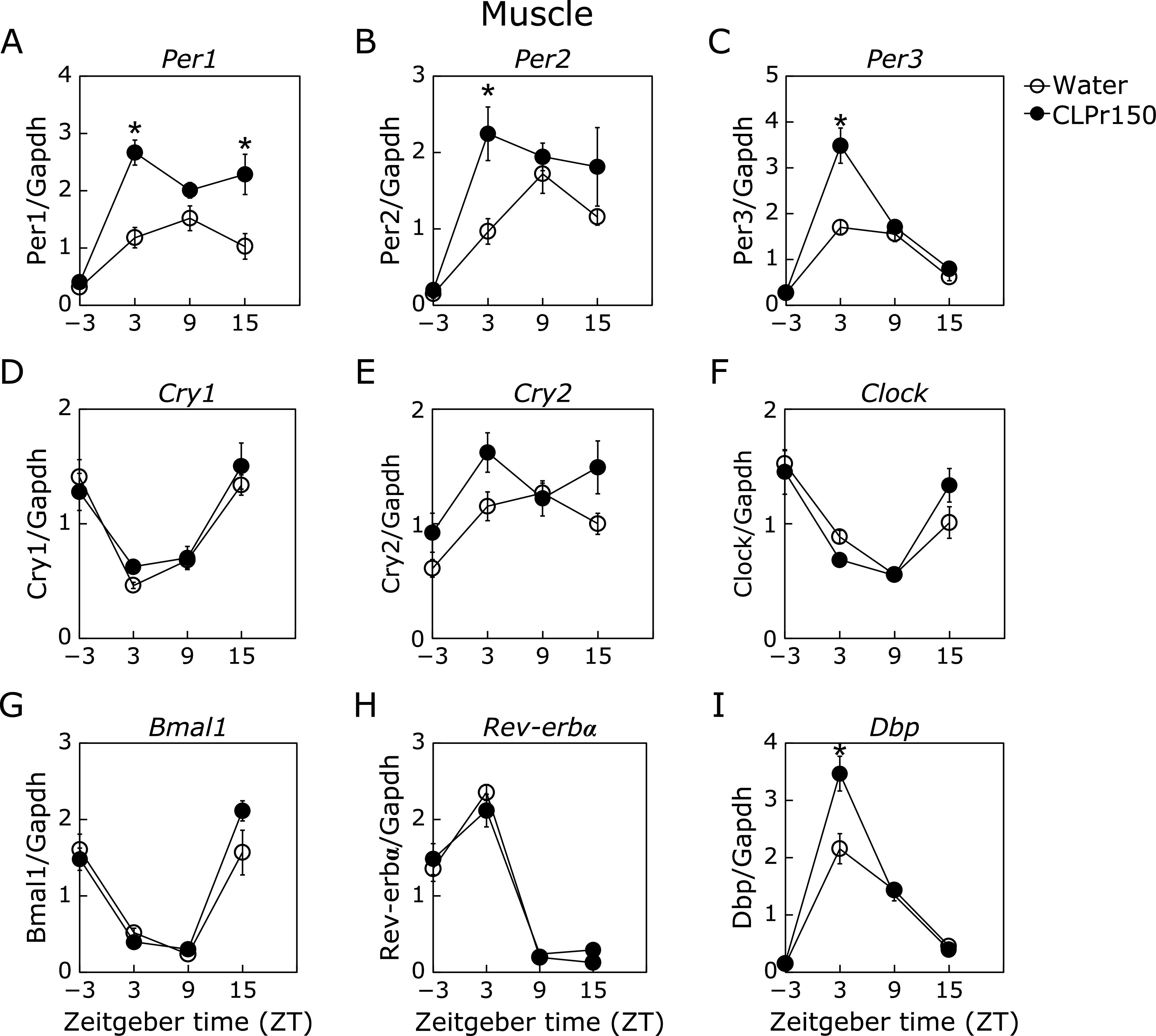
Expression of clock genes and energy metabolism- related genes in the muscle 3 h after the administration of CLPr. Mice were orally administered CLPr at 150 mg/kg body weight or water (5.0 ml/kg body weight) at ZT-3, 3, 9 or 15. The expression level of clock genes was measured in the muscle and their mRNA level was normalized by *gapdh*. The polygonal line graphs for *Per1* (A), *Per2* (B), *Per3* (C), *Cry1* (D), *Cry2* (E), *Clock* (F), *Bmal1* (G), *Rev-erbα* (H) and *Dbp* (I) are shown. Data are represented as the means ± SE (*n* = 5). *Significantly different from the control group at each time point (*p*<0.05; Dunnett’s test).

**Fig. 3:**
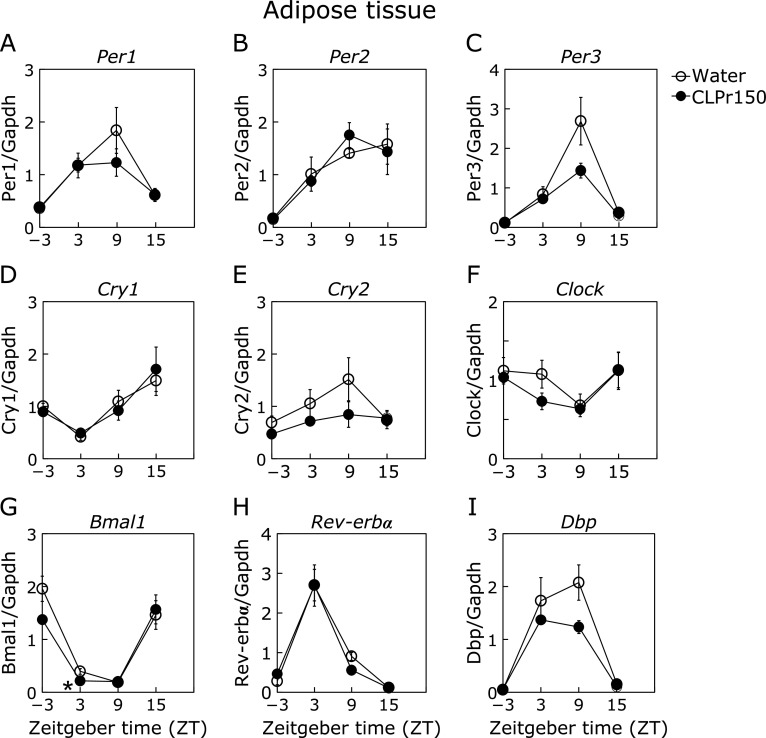
Expression of clock genes and energy metabolism-related genes in the white adipose tissue 3 h after the administration of CLPr. Mice were orally administered CLPr at 150 mg/kg body weight or water (5.0 ml/kg body weight) at ZT-3, 3, 9 or 15. The expression level of clock genes was measured in the white adipose tissue and their mRNA level was normalized by *gapdh*. The polygonal line graphs for *Per1* (A), *Per2* (B), *Per3* (C), *Cry1* (D), *Cry2* (E), *Clock* (F), *Bmal1* (G), *Rev-erbα* (H) and *Dbp* (I) are shown. Data are represented as the means ± SE (*n* = 5). *Significantly different from the control group at each time point (*p*<0.05; Dunnett’s test).

**Fig. 4:**
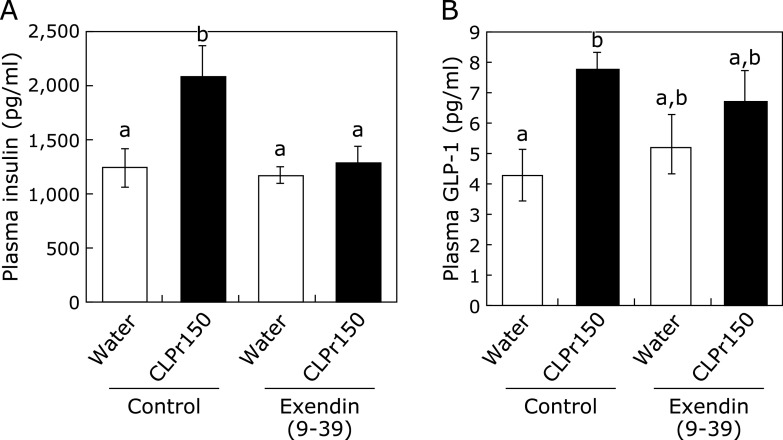
The effect of CLPr administration on the plasma level of GLP-1 and insulin after pretreatment with GLP-1 receptor antagonist. Mice were orally administered CLPr at 150 mg/kg body weight or water (5.0 ml/kg body weight) at ZT3. Exendin (9-39), a GLP-1 receptor antagonist, was pre-injected to the mice at 200 nmol/kg body weight 5 min before the CLPr administration. Plasma level of insulin and GLP-1 was measured by corresponding ELISA kit 1 h after the CLPr administration. Data are represented as the means ± SE (*n* = 5). Different letters indicate significant differences (*p*<0.05 by Tukey-Kramer test).

**Fig. 5:**
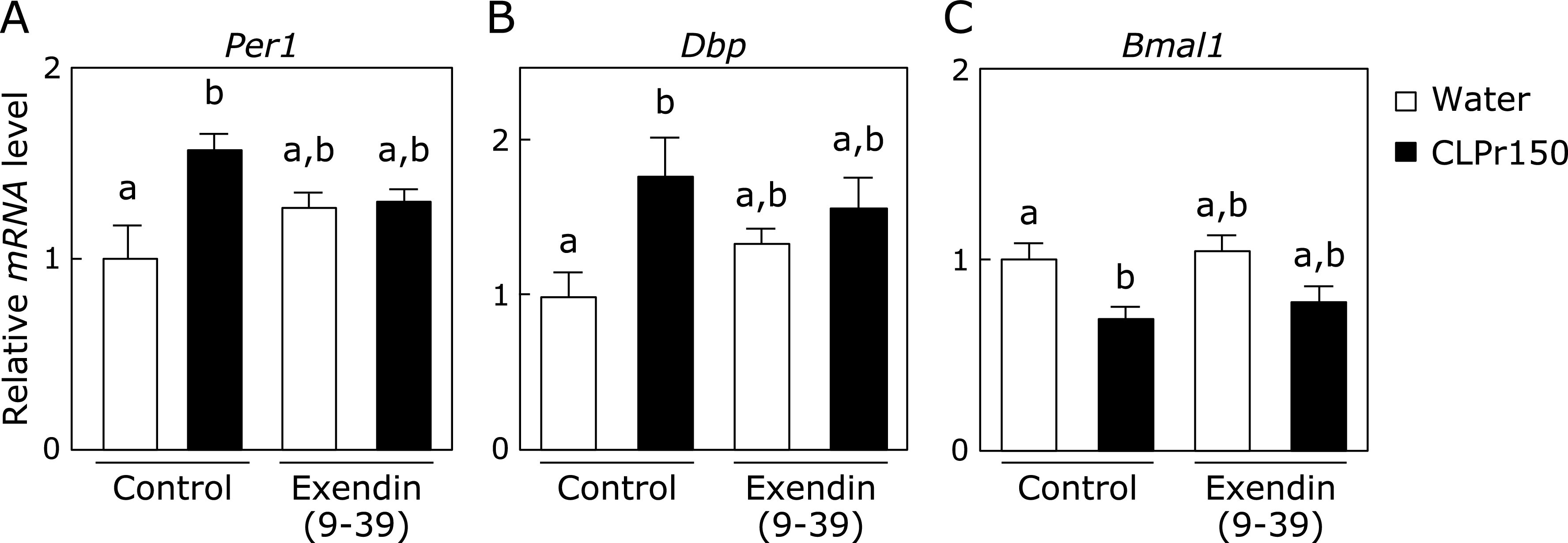
The effect of CLPr administration on the expression level of clock genes in the liver after pretreatment with GLP-1 receptor antagonist. Mice were orally administered CLPr at 150 mg/kg body weight or water (5.0 ml/kg body weight) at ZT3. Exendin (9-39), a GLP-1 receptor antagonist, was pre-injected to the mice at 200 nmol/kg body weight 5 min before the CLPr administration. The liver was collected 3 h after the CLPr administration and the expression of *Per1* (A), *Dbp* (B) and *Bmal1* (C) was measured by RT-PCR. After the expression level of mRNA was normalized by that of *gapdh*, the relative expression level was shown. Data are represented as the means ± SE (*n* = 5). Different letters indicate significant differences (*p*<0.05 by Tukey-Kramer test).

**Fig. 6:**
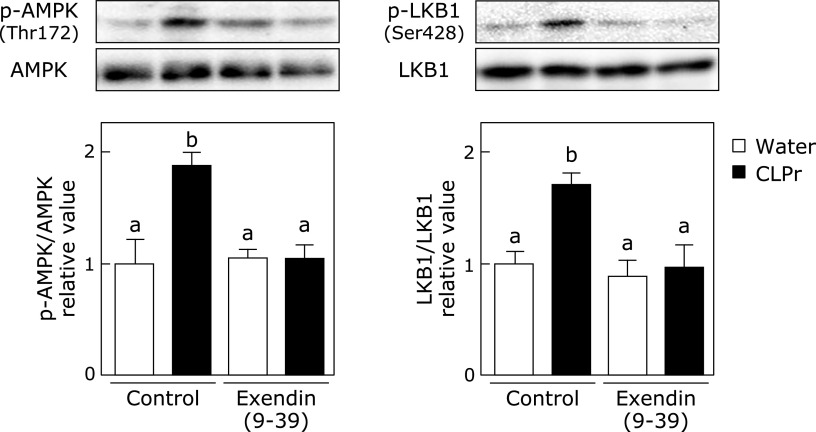
Effect of CLPr administration on the phosphorylation of AMPK and LKB1 after pretreatment with GLP-1 receptor antagonist. Mice were orally administered CLPr at 150 mg/kg body weight or water (5.0 ml/kg body weight) at ZT3. Exendin (9-39), a GLP-1 receptor antagonist, was pre-injected to the mice at 200 nmol/kg body weight 5 min before the CLPr administration. The liver was collected 1 h after the CLPr administration and phosphorylation of AMPK and LKB1 was measured the by western blotting. Density of the phosphorylation protein was normalized by that of corresponding expression protein. Data are represented as the means ± SE (*n* = 5). Different letters indicate significant differences (*p*<0.05 by Tukey-Kramer test).

The data are presented as the means ± SE. Dunnett multiple comparison test (Fig. 1, 5 and 6) or Tukey-Kramer multiple comparison test (Fig. 2–4) was used to determine the significant difference. The level of statistical significance was set to *p*<0.05.

## Results

### Effect of single administration of CLPr on the expression of clock genes at four different time-points in the peripheral tissues

In the experiment 1, it was investigated whether a single oral administration of CLPr at ZT-3, 3, 9 and 15 affected the expression rhythm of clock genes in liver, skeletal muscle and adipose tissue. In the liver, CLPr administration at ZT3 increased the expression level of *Per1*, *Per2*, *Per3* and gene for d-site of albumin promoter binding protein (*Dbp*) (Fig. 1A–C and I), while decreased that of *Bmal1* (Fig. 1G). In addition, CLPr administration at ZT15 increased the expression level of *Per1*, *Cry1* and *Bmal1* (Fig. 1A, D and G). CLPr administration at ZT-3 and ZT9 did not affect the expression of clock genes. In the muscle, CLPr administration at ZT3 also increased the expression levels of *Per1*, *Per2*, *Per3* and *Dbp* as the same trend as the results in the liver (Fig. 2). On the other hand, CLPr administration did not affect the expression of clock genes in the perirenal adipose tissue, except *Bmal1* at ZT3 (Fig. 3).

### CLPr regulates the circadian rhythm of clock gene expression through GLP-1 secretion in the liver

In the experiment 2, to evaluate whether CLPr increased GLP-1 and insulin secretion, the mice were orally administered of CLPr at 150 mg/kg body weight at ZT 3 and measured the concentration of GLP-1 and insulin in the plasma of the mice 1 h after the CLPr administration. As shown in Fig. 4, plasma GLP-1 and insulin concentrations were significantly increased. When GLP-1 receptor antagonist Exendin (9-39) was pretreated to the mice 5 min before the CLPr administration, it completely canceled the CLPr-increased plasma insulin concentration to the control level. On the other hand, Exendin (9-39) did not affect plasma GLP-1 concentration in both CLPr and control groups as expected because of the antagonist of GLP-1 receptor, but not GLP-1 inhibitor.

We further examined whether Exendin (9-39) canceled CLPr-altered the expression of clock genes in the liver 3 h after the CLPr administration at ZT3. As shown in Fig. 5, significant changes in the expression of *Per1*, *Dbp* and *Bmal1* by the CLPr administration were disappeared by pre-administration of Exendin (9-39). These results indicated that CLPr-increased GLP-1 secretion was involved in the altered expression of clock genes.

### CLPr regulates AMPK phosphorylation through GLP-1 at ZT3 in the liver

In the previous study, we have demonstrated that CLPr promotes phosphorylation of AMPK in the liver, muscle and adipose tissue.^(26)^ As shown in Fig. 6, phosphorylation of AMPK and its upstream kinase LKB1 in the liver was confirmed 1 h after the CLPr administration at ZT3 with or without injection of Exendin (9-39). As another upstream event of AMPK, phosphorylation of CaMKKβ was also measured, but it did not show any change. It was noteworthy that Exendin (9-39) canceled the CLPr-promoted phosphorylation of AMPK and LKB1. These results suggested that GLP-1 was also involved in CLPr-promoted phosphorylation of AMPK.

## Discussion

Most organisms have circadian rhythm to adjust their metabolic homeostasis for adaptation to the environment. Circadian rhythm is regulated by clock genes, especially the core clock ones such as *Per*, *Cry*, *Bmal1* and *Clock*.^(31)^ Feeding behavior and light stimulation regulate amplitude and phase of the clock gene expression.^(3)^ Disruption of the circadian rhythm lead to metabolic disorders such as type 2 diabetes and obesity.^(32)^ Recently, it has been reported that functional food ameliorates metabolic syndrome associating with circadian clock.^(33,34)^ With regard to CLPr, it prevents obesity and hyperglycemia.^(26,27)^ In this study, we found that CLPr regulated the circadian clock gene expression through GLP-1 signaling pathway in the liver using a GLP-1 receptor antagonist Exendin (9-39) (Fig. 1 and 5). GLP-1 also regulated CLPr-induced activation of LKB1/AMPK pathway (Fig. 6). This is the first report that the GLP-1 receptor antagonist affected the expression level of clock genes and the GLP-1 was involved in phosphorylation of AMPK promoted by procyanidin-rich CLPr. These previous and current results suggest that the clock gene expression is deeply involved in the prevention mechanism of CLPr on metabolic disorders and that there exists an effective and suitable timing for an intake of procyanidin rich foods to get the health beneficial functions. Liver plays a central role in metabolism and energy utilization and regulates the physiological status of the whole body. It is known that the liver is the most vital zeitgeber organ for peripheral clocks.^(35)^ Results in this study showed that CLPr administration at ZT3 increased expression level of *Per1*, *Per2*, *Per3* and *Dbp* in the liver (Fig. 1), indicating that the liver is responsible for the alteration of clock gene expression by CLPr administration. Clock genes in the muscle also weekly respond to CLPr (Fig. 2), but those in adipose tissue showed almost no response against CLPr (Fig. 3). Thus, the liver is the most susceptible tissue among examined three tissues after the intake of procyanidin rich foods. In the liver, it was reported that insulin directly regulates the phase entrainment of circadian oscillators, especially at the light phase: An injection of insulin at the light phase advanced the phase of circadian rhythm and increased the expression of *Per1*, *Per2*, *Per3* and *Dbp* through mitogen-activated protein kinase, phosphoinositide 3-kinase and protein kinase Cα in the liver of mice.^(14,36)^ In the present study, it was confirmed CLPr administration increased plasma insulin and GLP-1 concentrations (Fig. 4) as the same manner as our previous report.^(37)^ In addition, a GLP-1 antagonist Exendin (9-39) completely inhibited CLPr-increased plasma insulin (Fig. 4) and attenuated CLPr-altered expression of *Per1*, *Dbp* and *Bmal1*. Therefore, the alteration of clock genes by CLPr is explainable due to the increased insulin through CLPr-increased secretion of GLP-1. These findings may be involved the improvement of hyperglycemia and energy metabolism by CLPr administration. However, we did not address whether obtained findings were dependent on the phase advance of circadian rhythm by insulin. To clarify this issue, it is necessary to perform more experiments in future.

Recently, it has been reported that GLP-1, which is released from the gut L cells, is involved in not only secretion of insulin from pancreatic β-cells, but also modulation of the metabolisms and energy expenditure.^(38)^ In this study, the GLP-1 receptor antagonist Exendin (9-39) completely canceled CLPr-caused phosphorylation of AMPK (Fig. 6). This finding supports previous one that an agonist of GLP-1 receptor liraglutide promoted AMPK phosphorylation in the liver.^(39)^ AMPK is an important regulator of metabolism and circadian rhythm.^(40)^ It is reported that AMPK regulates the expression of several clock gene products in mammalian tissues, because AMPK phosphorylates CRY1 and CRY2 and stimulates their degradation.^(41)^ However, we could not observe CRY family expressions by CLPr administration. These results suggest that changes in the clock gene expression by the CLPr administration at ZT3 is independent of AMPK phosphorylation.

CLPr-increased plasma insulin and GLP-1 levels are involved in the decreased expression of *Bmal1* at ZT3, because it is reported that the circadian rhythm of *Bmal1* is in parallel with the plasma insulin and GLP-1 levels.^(42)^ Function of *Bmal1* in the liver is important to buffering the circulating glucose level in a time-of-day dependent manner, and contributes to systemic glucose homeostasis.^(43)^ On the other hand, we previously reported that a single oral administration of CLPr suppressed postprandial hyperglycemia through both GLP-1 and AMPK pathways. It is known that AMPK and its downstream factor peroxisome proliferator-activated receptor gamma coactivator 1α (PGC-1α) are involved in the decrease in *Bmal1* expression.^(44)^ Although PGC-1α expression increased at ZT3 (data not shown), AMPK pathway might not associate with CLPr-caused changes in the clock gene expression as above mentioned. It is, therefore, suggested that CLPr-caused down-regulation of *Bmal1* may be involved in amelioration of postprandial hyperglycemia.

Currently, researches in the field of chrono-nutrition are progressing rapidly. It is increasing recognition on the importance of the interaction between food components and expression of circadian clock. Certain polyphenols such as resveratrol, nobiletin and proanthocyanidins possess chronobiological properties and can affect the expression of peripheral clock genes. For example, resveratrol affects the circadian rhythms by changing the phase shift of *Bmal1*.^(18,19)^ Nobiletin protects against insulin resistance and disorders of lipid metabolism through regulation of *Bmal1* expression in hepatocytes.^(45)^ The same report demonstrated that nobiletin regulates not only expression of Bmal1 but also phosphorylation of AMPK. Bmal1 and AMPK are the key regulators for metabolic fitness by regulating the hepatic mitochondrial function.^(45)^ In addition, certain nutrients increase *Per2* expression through the insulin secretion.^(33,34)^ Taken together findings in this study and these previous reports suggest that CLPr acts as a clock-regulator to prevent and/or ameliorate metabolic disorders when administration of it at the suitable timing.

In conclusion, in this study, we found that CLPr affected the expression of circadian clock genes *Per1*, *Per2*, *Per3* and *Bmal1* and promoted secretion of GLP-1 and phosphorylation of AMPK. These findings indicate that CLPr is a possible functional food material for prevention and/or amelioration of metabolic disorders through preventing circadian disruption by activating GLP-1 and AMPK pathways.

## Figures and Tables

**Table 1:** Composition of polyphenol in cacao liquor extract (CLPr)

Composition	% (w/w)
Catechin	4.28
Epicatechin	6.12
Procyanidin B2	3.6
Procyanidin B5	0.75
Procyanidin C1	2.28
Cinnamyannin A2	1.01

Total polyphenol	69.8

**Table 2:** Primer sequences used for real-time PCR amplification

Gene	Forward primer	Reverse primer
Gapdh	catggccttccgtgttccta	cctgcttcaccaccttcttga
Per1	gcttcgtggacttgacacct	tgctttagatcggcagtggt
Per2	caacacagacgacagcatca	tcctggtcctccttcaacac
Per3	gtgaagccagtggcagaga	tgagaggaagaaaagtccttctg
Cry1	atcgtgcgcatttcacatac	tccgccattgagttctatgat
Cry2	gcagagcctggttcaagc	ggccactggatagtgctctg
Clock	ccagtcagttggtccatcatt	tggctcctaactgagctgaaa
Bmall	tcagatgacgaactgaaacacc	cggtcacatcctacgacaaa
Rev-erbα	ccctggactccaataacaaca	tgccattggagctgtcact
Dbp	gcattccaggccatgagact	ccagtacttctcatccttctgt

## Abbreviations

AMPK: AMP-activated protein kinase
BMAL1: brain-muscle arnt like protein 1
CaMKK: Ca^2+^/calmodulin-dependent protein kinase kinase
CLOCK: circadian locomotor output cycle kaput
CLPr: cacao liquor procyanidins extract
CRY: cryptochrome
DBP: d-element-binding protein
ELISA: enzyme-linked immunosorbent assay
GLP-1: glucagon like peptide-1
LKB: live kinase B1
PER: period
Rev-erbα: reverse erythroblastosis virus alpha
ZT: zeitgerber time
